# Prickly Pear and Fibromyalgia: A Conceptual Protocol for Plant-Based Symptom Management

**DOI:** 10.3390/nu17213441

**Published:** 2025-10-31

**Authors:** Orly Sarid, Orli Grinstein-Cohen, Noemi Tel-Zur

**Affiliations:** 1 The Spitzer Department of Social Work, Ben-Gurion University of the Negev, Beer-Sheva 84105, Israel; orlysa@bgu.ac.il; 2 Goldman Sonnenfeldt School of Sustainability and Climate Change, Ben-Gurion University of the Negev, Beer-Sheva 84105, Israel; 3 Faculty of Health Sciences, Ben-Gurion University of the Negev, Beer-Sheva 84105, Israel; grinstie@bgu.ac.il; 4 French Associates Institute for Agriculture and Biotechnology of Drylands, J. Blaustein Institutes for Desert Research, Ben-Gurion University of the Negev, Beer-Sheva 84105, Israel

**Keywords:** clinical research, fibromyalgia syndrome (FMS), fruit consumption, prickly pear

## Abstract

Nutrition is being increasingly recognized as a modifiable adjuvant factor in symptom management, yet few studies have examined the direct contribution of fruit consumption to chronic disease outcomes. The existing research largely emphasizes broad dietary patterns or isolated nutrients, rather than specific fruit species and their complex bioactive profiles. This gap is particularly evident in conditions lacking disease-specific pharmacological treatments, such as fibromyalgia syndrome (FMS), where patients often depend on lifestyle adjustments and complementary strategies for symptom relief. The therapeutic use of fruits presents methodological challenges, as their biochemical composition is strongly influenced by abiotic and biotic stresses, seasonal and regional variations, as well as post-harvest handling and storage. Such variability complicates reproducibility and obscures causal links in clinical research. While reductionist approaches that isolate single compounds offer dose control, they risk losing synergistic effects inherent to whole fruits. Conversely, whole-fruit consumption preserves integrative complexity but introduces variability. Overcoming these limitations requires rigorous standardization across agricultural, nutritional, and clinical domains, accurate species and cultivar identification, controlled cultivation conditions, chemical fingerprinting, and biomarker validation. In this context, cacti fruits such as *Opuntia ficus-indica* (prickly pear), which is rich in betalains and polyphenols, emerge as promise adjuvant agents for FMS symptom management. We propose a protocol designed to systematically evaluate their efficacy and feasibility in clinical application, aiming to strengthen the reliability and accuracy of research outcomes.

## 1. Introduction

Although nutrition is increasingly recognized as a modifiable adjuvant factor in symptom management, only a few studies have directly examined the links between fruit consumption and chronic diseases. Most available studies focus broadly on dietary patterns, such as the Mediterranean diet, or on isolated nutrients such as fiber or polyphenols, rather than on specific fruit species and their potential immunoregulatory properties [1]. This gap is particularly striking in conditions that lack disease-specific pharmacological treatments, such as fibromyalgia syndrome (FMS) where patients often rely on lifestyle adjustments and complementary approaches to manage symptoms [2]. In these contexts, exploring the role of fruits with their complex mixtures of bioactive compounds represents an underdeveloped but potentially valuable avenue for symptom relief.

A major methodological challenge in utilizing fruits for therapeutic purposes arises from [3] the fact that plants are sessile organisms, which are cultivated under non-controlled conditions and are consequently exposed to abiotic stresses such as drought, extreme temperatures, and high solar radiation, as well as biotic stresses including pathogen attack [3,4]. These stress factors substantially reprogram fruit metabolism, leading to shifts in metabolite profiles, enhanced biosynthesis of protective antioxidants, and induction of stress-responsive pigments [5,6]. As a result, the concentrations of vitamins, phenolics, and other secondary metabolites can fluctuate significantly across seasons and growing regions [7,8]. Moreover, post-harvest handling and storage conditions critically influence antioxidant stability and modulate the sugar-acid equilibrium in fruits [9,10]. Moreover, the biochemical composition of fruits is highly sensitive to environmental factors, such as soil quality and irrigation [11,12], as well as to the harvest time, the cultivar and the genetic background of the crop [13,14]. Such variability introduces substantial noise into clinical studies, as patients may consume fruits with differing bioactive compound contents. This variability complicates the attribution of observed outcomes specifically to fruit intake. Without rigorous control and precise reporting of cultivar, ripeness stage, harvest conditions, and biochemical composition, studies risk producing biased estimates that may either underestimate or overstate the true therapeutic potential of a given fruit species. However, it is important to remark that reducing plants to isolated compounds, as in the case of standardized cannabis derivatives, provides dose control but risks losing the synergistic “entourage effect” or food synergy arising from multiple interacting bioactive [15,16]. Conversely, consuming the whole fruit preserves this integrative complexity but introduces uncontrolled variability that undermines reproducibility in clinical research. For this reason, international guidelines emphasize the need for rigorous horticulture standardization, including species identification, cultivation conditions, harvest indices, chemical fingerprinting, and exposure biomarkers [17]. Addressing these limitations requires integrating agricultural, nutritional, and medical aspects to establish standardized protocols for plant material used in human disease trials. This would involve biochemical fingerprinting of fruits before use, strict documentation of harvest and storage variables, and validation of participant self-reports. Only through such methodological rigor can the therapeutic potential of fruits as adjunctive interventions for symptom management be reliably and reproducibly assessed.

## 2. Epidemiological Overview of Fibromyalgia Syndrome (FMS) and Nutraceutical Use

Fibromyalgia syndrome affects approximately 2–4% of the adult population worldwide, with higher prevalence among women [18]. Clinical registries in North America and Europe report that women constitute 80–90% of diagnosed cases, though recent analyses suggest that men may be underdiagnosed due to referral and diagnostic bias [19]. The hallmark symptoms of FMS include chronic widespread musculoskeletal pain, persistent fatigue, unrefreshing sleep, cognitive dysfunction, and frequent mood disturbances, often clustering in ways that amplify disability [20,21]. These symptoms impose a considerable burden on daily functioning and quality of life [22].

Pharmacologic treatments for managing FMS, including antidepressants, anticonvulsants, and analgesics, remain central, with pregabalin, duloxetine, and milnacipran being the only FDA-approved medications. However, their effectiveness is limited and varies among patients [23,24]. Combining pharmacological and nutraceutical treatments is a viable therapeutic option to effectively manage the daily symptoms of patients with FMS. This integrative approach emphasizes the importance of creating individualized treatment plans that consider each patient’s unique symptoms, needs, and tolerances [21]. Consequently, nutraceuticals have been increasingly explored as adjunctive interventions, among them is coenzyme Q10, which has shown potential to alleviate pain and fatigue by enhancing mitochondrial function and attenuating inflammatory pathways [25]. Other micronutrients and antioxidants are magnesium, vitamin B12, alpha-lipoic acid, melatonin, iron, probiotics, curcumin, and omega-3 fatty acids, with some studies reporting benefits across sleep, mood, gastrointestinal symptoms, and overall quality of life, although the findings remain inconsistent [2].

Emerging candidates for FMS management include palmitoylethanolamide (PEA), an endogenous fatty acid amide with documented anti-inflammatory activity, and polyphenol-rich extracts, which have demonstrated analgesic and neuroprotective effects in preclinical models and early-phase clinical studies [26]. Small, randomized trials further suggest that nutraceutical blends, such as olive-based supplements combined with anti-inflammatory dietary strategies, may reduce pain and fatigue severity in women with FMS, while comparative studies explored nutraceuticals alongside acupuncture and other complementary therapies [27]. Emerging evidence also points toward cannabis-derived compounds and low-dose naltrexone (LDN) as promising adjuncts, though they remain outside standard nutraceutical classification and require further validation [28].

## 3. Cacti and FMS

Rising temperatures, erratic rainfall, prolonged droughts, and increasing soil salinity disproportionately affect arid and semi-arid regions, exacerbating the hardships of populations already struggling with poverty and food insecurity. Cacti, particularly *Opuntia ficus-indica*, known as prickly pear, represent one of the most resilient crops in the context of climate change. Their physiology is adapted to arid and semi-arid environments, making them especially valuable in marginal agricultural regions affected by desertification [29,30], which are expected to expand due to rising temperatures, irregular rainfall, and increasing soil salinity. A central feature of their resilience is Crassulacean Acid Metabolism (CAM) photosynthesis, which allows stomata to remain closed during the day and open at night, reducing water loss by up to 80% compared with C3 plants [31,32]. This adaptation enables cacti to maintain productivity under prolonged drought and high solar radiation, conditions that severely reduce yields of conventional crops [31].

Preclinical and human evidence has demonstrated that prickly pear exerts systemic anti-inflammatory effects, largely through its betalain pigments (betanin, indicaxanthin) [33,34]. These compounds downregulate pro-inflammatory cytokines such as TNF-α, IL-6, and IL-1β, which are central to nociceptor sensitization and immune-driven pain amplification [35].

Of particular relevance is indicaxanthin, the dominant yellow betaxanthin in prickly pear [36]. Unlike many plant pigments, indicaxanthin crosses the blood–brain barrier [37,38,39]. Within neural tissue it modulates glutamatergic neurotransmission and maintains redox balance, suggesting a potential role in reducing central sensitization, a key mechanism in FMS. Therefore, indicaxanthin may represent a candidate molecule for linking peripheral dietary intake to central modulation of pain.

In a previous study, we investigated the effects of daily prickly-pear consumption in women with FMS living in dryland environments where stressors and seasonal variability are particularly pronounced. Our findings indicated that twice-a-day prickly-pear intake was associated with significant reductions in reported pain severity, accompanied by improvements in quality-of-life indices [40]. These results highlight prickly pear as a potential adjunctive dietary strategy for managing pain in FMS.

From a nutraceutical perspective, cacti fruits contain soluble fibers, mucilage polysaccharides, vitamins, minerals, and unique pigments such as indicaxanthin, which exert prebiotic, antioxidant, and anti-inflammatory effects [41,42,43,44]. These properties are particularly significant for populations in low-resource settings, where access to pharmaceuticals for chronic illnesses such as FMS is limited, and affordable dietary interventions may provide meaningful symptom relief.

**Figure 1:**
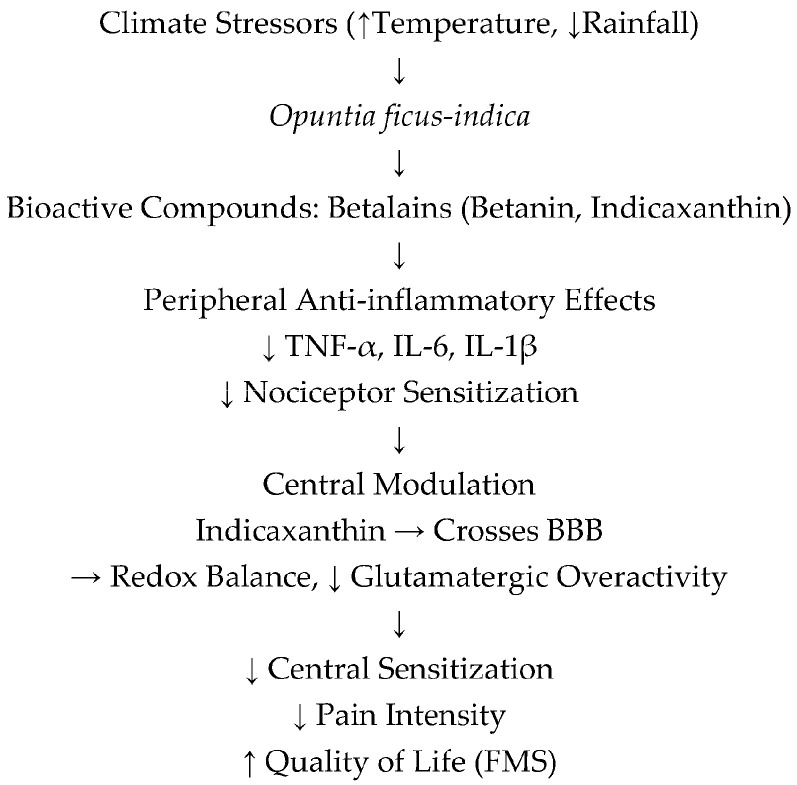
Bioactive compounds of prickly pear and their proposed roles in modulating pain in fibromyalgia.

Nutraceuticals represent an appealing strategy for FMS symptom relief. However, a central methodological challenge is the absence of regulation and standardization in the use of fruits for therapeutic purposes. The biochemical profile of fruits is influenced not only by cultivar, soil composition, irrigation regime, agronomic practices, and ripening stage, but also by the daily physiological rhythm characteristic of CAM species such as cacti [45]. The central challenge lies in balancing the affordability and availability of prickly pear with rigorous standardization, thereby ensuring that its therapeutic benefits are supported by reproducible evidence rather than confounded by uncontrolled natural variability. The bioactive compounds of prickly pear, proposed to modulate pain in fibromyalgia via anti-inflammatory, antioxidant, and neuromodulator effects, are summarized in Figure 1.

## 4. Standardizing Prickly Pear for Clinical Research

Here, we propose a five-step protocol encompassing essential procedures to standardize the biochemical composition of prickly pear fruits, thereby ensuring consistency, reproducibility, and reliability in clinical investigations.

The first step addresses fruit composition and quality parameters. Prickly pear fruit is particularly rich in yellow indicaxanthin. Bioactive compounds such as indicaxanthin and other betaxanthins, along with soluble fiber and mucilage, should be quantified using HPLC or LC-MS methods and reported in mg/g dry weight, since these are the components most directly linked to anti-inflammatory effects [35,37,41,42]. In addition, the broader phenolic profile and vitamin C and E, sugar, and organic acid contents should be determined, as these influence both biological activity and tolerability [46,47]. Cultivar and ripeness are the primary determinants of the fruit biochemical composition. Ripening can be standardized through Brix (sugar content), titratable acidity, pH, and external color index, all of which correlate with metabolite balance and consumer quality [46,48,49]. For fruit, agronomic practices, such as irrigation quality (for example greywater), pesticide or fungicide application, fertilization, shading, and pruning, all may alter metabolite composition and introduce confounding residues or stress responses [50,51]. Importantly, the metabolite profile of prickly pear fruit differs significantly between cultivars [52]. Yellow, red, purple, and white varieties vary in concentrations of betacyanins, betaxanthins, ascorbic acid, polyphenols, and carotenoids [41,52,53,54,55]. For example, red cultivars are generally richer in betacyanins, while yellow cultivars contain higher levels of indicaxanthin, which has been shown to cross the blood–brain barrier [39,56]. These cultivar-specific differences directly affect antioxidant potential, and biological activity, underscoring the need for cultivar-level specification in any nutraceutical or clinical application. Finally, safety and stability parameters, including microbial load, pesticide residues, drying temperature, packaging atmosphere, and cold storage, must be documented [42,56,57].

The second step concerns harvest timing. Environmental conditions also shape fruit composition. High solar radiation, elevated temperatures, and drought stress increase the synthesis of betalains, flavonoids, and phenolic acids as part of the plant’s stress defenses, thereby enhancing its antioxidant capacity [58].

In the cladode of *Opuntia ficus-indica*, daily metabolic rhythms have been documented: at night, malic acid and other organic acids accumulate through CAM, whereas during the day these acids are decarboxylated and metabolized, leading to diurnal shifts in titratable acidity and the sweetness-to-acid ratio [59,60]. Cladodes picked in the morning tend to contain higher levels of organic acids, whereas those harvested later in the day are sweeter and exhibit altered antioxidant profiles [61]. These fluctuations not only alter the flavor but also affect the chemical stability and potential bioavailability of bioactive compounds. However, in the absence of data on daily metabolic rhythms in fruits, standardizing the harvest time is critical to ensure consistency in biochemical composition and reproducibility in clinical studies.

The third step addressed post-harvest treatments. Storage and handling conditions as equally important: temperature, humidity, acidity, and duration after harvest influence indicaxanthin stability, fruit firmness, enzymatic activity, and oxidative degradation. Post-harvest management is therefore a key determinant of prickly pear fruit quality [46,62,63]. While these factors may not be tightly controlled in all settings, transparent reporting allows researchers to account for their influence in analyses. For example, Cruz-Bravo et al. [64] showed that cold storage of prickly pear fruit preserves bioactive compounds and antioxidant activity, highlighting the importance of standardized post-harvest conditions for ensuring consistent exposure to clinical interventions, even though it may reduce fruit mass. Taken together, these findings indicate that the chemical profile of prickly pear fruit is not fixed but reflects the interaction of cultivar genetics, predictable physiological rhythms, pre-harvest environmental stress, and post-harvest handling. For experimental and clinical studies, rigorous documentation of each batch is essential to ensure reproducibility, accurate dosing, and reliable interpretation of outcomes.

The fourth step addresses FMS patients. In FMS, there are no reliable objective biomarkers or laboratory tests that can confirm the diagnosis or monitor the disease severity. The syndrome is defined by subjective symptoms such as widespread pain, fatigue, cognitive complaints (“fibro fog”), and reduced physical and occupational functioning. Because no specific biological marker has been fully validated [65], assessment often relies on patient self-report questionnaires that capture symptom severity and its impact on daily life [66]. The key domains to evaluate include pain, commonly measured by the Visual Analogue Scale (VAS) or the Brief Pain Inventory; fatigue, often assessed with the Multidimensional Fatigue Inventory (MFI) or Fatigue Severity Scale (FSS); quality of life, measured by instruments such as the SF-36 or the Fibromyalgia Impact Questionnaire (FIQ); and functioning and work ability, captured by tools like the Work Productivity and Activity Impairment Questionnaire (WPAI). These scales are important because they reflect the patient’s lived experience of FMS, integrating pain intensity, physical and social role limitations, and overall well-being dimensions that cannot be observed through imaging, blood tests, or physical examination alone [2,66].

Symptom assessment in FMS should take into account circadian patterns, as pain and fatigue are closely linked to daily physiological rhythms. Morning nadirs in cortisol have been associated with greater stiffness and pain, whereas evening peaks often reflect the cumulative effects of activity and stress throughout the day [67,68]. Patient assessments should be anchored at fixed morning and evening times. However, requiring patients to complete comprehensive scales twice a day would likely reduce adherence and increase respondent burden. A pragmatic approach is to administer the full battery at baseline and at the end of the study. Validated instruments include the Fibromyalgia Impact Questionnaire–Revised (FIQ-R) for overall functional impact, the Brief Pain Inventory (BPI) for pain intensity and interference, and the Pittsburgh Sleep Quality Index (PSQI) for sleep disturbances [69,70]. For depressive symptoms, the Hospital Anxiety and Depression Scale (HADS) is recommended as it minimizes the confounding influence of somatic overlap with FMS-related symptoms [71].

Brief daily assessments to capture morning and evening states can be collected with ultra-brief items rated on numeric scales: subjective units of distress (SUDS) [72,73]; fatigue using a validated single-item fatigue measure [71]; and pain intensity using the Visual Analogue Scale (VAS), a reliable and valid single-item measure that offers high sensitivity and discrimination in repeated assessments [74].

In settings where literacy barriers exist, standard patient-reported questionnaires may need to be adapted. Visual analog tools using graded color scales (e.g., from light to dark shades representing increasing pain or fatigue intensity) or simple numeric sequences can provide intuitive, culturally neutral means to capture subjective experiences. Such adaptations have been successfully applied in low-literacy populations and may enhance the inclusiveness and validity of symptom assessment within the proposed protocol. In a previous study [75] conducted among outpatients with acute and chronic burn injuries, it was demonstrated that even in contexts with limited linguistic or communicative resources, simple subjective assessment tools can yield valid and clinically meaningful measures of pain and distress.

The fifth step addresses FMS patients and cacti intake. Aligning the timing of nutraceutical intake with the symptom rhythms of FMS patients may enhance therapeutic benefit. Pain and fatigue often peak in the morning due to poor restorative sleep and cortisol dysregulation [76,77]. Thus, morning consumption of prickly pear may be advantageous to counteract oxidative and inflammatory stress at the time of greatest symptom intensity. Conversely, evening supplementation may help reduce oxidative stress before sleep, potentially improving recovery and sleep quality.

Seasonal patterns are also important to consider. Many patients report worsening symptoms during colder months, consistent with evidence that low ambient temperatures exacerbate musculoskeletal pain and fatigue [78]. Fruits harvested in summer, when their betalain and polyphenol content is at its peak, may therefore provide a stronger nutraceutical effect. If fruits can be frozen, this may extend their availability; however, studies are needed to evaluate the stability of bioactive compounds under freezing conditions to ensure consistency and efficacy in clinical interventions. Table 1 summarizes the five steps of the proposed standardization protocol for prickly pear in clinical research, outlining the key objectives, critical parameters to be controlled or measured, and methodological considerations for each step.

To minimize participant burden and enhance adherence, future studies can employ a dedicated mobile application for real-time symptom tracking. Participants would record pain, fatigue, and mood twice daily a simple 0–10 numeric scale, a method shown to be feasible and well-accepted in similar chronic illness contexts (e.g., Crohn’s disease monitoring apps) [79]. This digital approach allows standardized data collection with minimal effort and supports timely feedback and compliance monitoring.

## 5. Clinical Contraindications for Prickly Pear Fruit Consumption

Gastrointestinal disturbances are frequent in individuals with FMS, including nausea, bloating, altered bowel habits, and abdominal discomfort [80]. Introducing fruits with distinct digestive effects, such as prickly pear, may therefore directly provoke or indirectly intensify these symptoms. Although consumption of fresh fruit is generally considered safe, in FMS, the use of fruit as a nutraceutical may intensify gastrointestinal complaints and should be approached with caution. Moreover, evidence regarding safety during pregnancy and lactation remains insufficient, making medicinal or extract-based consumption inadvisable in these populations [40]. Given that some FMS patients may experience gastrointestinal sensitivity or mild renal dysfunction, future trials should include pre-screening for these conditions and apply exclusion criteria when necessary. Regular monitoring of gastrointestinal tolerance and renal function is recommended, particularly during prolonged intake. The use of seed-free prickly pear preparations may further reduce digestive discomfort while preserving the fruit’s bioactive benefits.

Patients with chronic kidney disease may also be at risk due to potential disruptions in potassium homeostasis and related electrolyte imbalances [81]. Furthermore, interactions with pharmacological agents, particularly antidiabetic and antihypertensive medications, should be considered [27,82], even though no direct contraindications have been reported with standard FMS treatments such as antidepressants and analgesics [68]. Taken together, these considerations highlight the necessity of individualized clinical evaluation of comorbidities and concomitant pharmacotherapy prior to recommending regular nutraceutical use of prickly pear [68].

## 6. Conclusions

This protocol establishes a structured framework for investigating prickly pear as an adjunctive nutraceutical in the management of FMS. By systematically addressing fruit composition and quality control, harvest timing, post-harvest handling, patient-level symptom assessments, and circadian alignment of intake, it provides the methodological precision needed to overcome the variability in nutraceutical studies. Standardization of indicaxanthin and other bioactive compounds through validated analytical methods (e.g., HPLC, LC-MS) ensures reproducible dosing, while integration of validated symptom scales with ultra-brief morning and evening assessments enhances sensitivity to daily fluctuations in distress, pain and fatigue. Future studies should aim to isolate and systematically evaluate the effects of specific bioactive compounds, particularly indicaxanthin, to elucidate their mechanistic contribution to pain modulation and central sensitization in FMS. Such work would require controlled clinical settings and standardized compound preparations to ensure reproducibility and safety. Despite these methodological challenges, this represents a promising direction for advancing the nutraceutical potential of *Opuntia ficus-indica*. Moreover, this conceptual framework does not control for all potential confounding factors influencing circadian symptom variability, such as sleep quality, medication timing, and daily activity levels. Future clinical studies should document these variables and include them as covariates in the analysis. Thus, the proposed timing of intake is designed to complement, rather than replace, patients’ standard treatments, which may vary across individuals and settings.

Taken together, this protocol creates the conditions for generating reproducible evidence on the therapeutic potential of *Opuntia ficus-indica* in FM.

## Figures and Tables

**Table 1:** A five-step protocol for the standardization of prickly pear in clinical research.

Step	Focus	Key Parameters/Procedures	Main Considerations
1. Fruit composition and quality	Quantification of bioactive compounds and quality traits	Indicaxanthin, betaxanthins, polyphenols, vitamins C/E, Brix, acidity, cultivar differences, safety parameters (microbial load, residues)	Ensure biochemical consistency and reproducibility
2. Harvest timing	Standardizing harvesting time and fruit maturation stage to control biochemical variability	Environmental stress, time of day, solar radiation, diurnal variation	Standardize picking time to minimize variability in metabolite content
3. Post-harvest treatments	Storage and handling conditions	Temperature, humidity, storage duration, cold preservation	To ensure reproducibility and minimize bioactive compound instability
4. FMS patient assessment	Outcome measures for clinical trials	Pain (VAS, BPI), fatigue (MFI, FSS), sleep (PSQI), quality of life (FIQ-R), depression (HADS)	Use validated instruments; align timing with circadian symptom patterns
5. Intake timing and seasonal aspects	Synchronizing supplementation with symptom rhythms	Morning vs. evening intake, seasonal variation, fruit availability	Optimize intake timing to match peak symptoms and enhance therapeutic effects

## Data Availability

No new data were created or analyzed in this study. Data sharing is not applicable to this article.
